# Insecticide resistance status of *Aedes aegypti* in southern and northern Ghana

**DOI:** 10.1186/s13071-023-05752-x

**Published:** 2023-04-18

**Authors:** Anisa Abdulai, Christopher Mfum Owusu-Asenso, Gabriel Akosah-Brempong, Abdul Rahim Mohammed, Isaac Kwame Sraku, Simon Kwaku Attah, Akua Obeng Forson, David Weetman, Yaw Asare Afrane

**Affiliations:** 1 Department of Medical Microbiology, Centre for Vector-Borne Disease Research, University of Ghana Medical School, University of Ghana, Accra, Ghana; 2 African Regional Postgraduate Program in Insect Science, University of Ghana, Legon, Accra, Ghana; 3 Department of Medical Laboratory Sciences, School of Biomedical and Allied Health Sciences, University of Ghana, Accra, Ghana; 4 Department of Vector Biology, Liverpool School of Tropical Medicine, Liverpool, UK

**Keywords:** Insecticide resistance, Target-site mutations, *Aedes aegypti*, Piperonyl butoxide synergist, Knockdown resistance, Ghana

## Abstract

**Background:**

Outbreaks of *Aedes*-borne arboviral diseases are becoming rampant in Africa. In Ghana, there is no organized arboviral control programme with interventions restricted to mitigate outbreaks. Insecticide application is a crucial part of outbreak responses and future preventative control measures. Thus, knowledge of the resistance status and underlying mechanisms of *Aedes* populations is required to ensure optimal insecticide choices. The present study assessed the insecticide resistance status of *Aedes aegypti* populations from southern Ghana (Accra, Tema and Ada Foah) and northern Ghana (Navrongo) respectively.

**Methods:**

Phenotypic resistance was determined with WHO susceptibility tests using *Ae. aegypti* collected as larvae and reared into adults. Knockdown resistance (*kdr*) mutations were detected using allele-specific PCR. Synergist assays were performed with piperonyl butoxide (PBO) to investigate the possible involvement of metabolic mechanisms in resistance phenotypes.

**Results:**

Resistance to DDT was moderate to high across sites (11.3 to 75.8%) and, for the pyrethroids deltamethrin and permethrin, moderate resistance was detected (62.5 to 88.8%). The 1534C *kdr* and 1016I *kdr* alleles were common in all sites (0.65 to 1) and may be on a trajectory toward fixation. In addition, a third *kdr* mutant, V410L, was detected at lower frequencies (0.03 to 0.31). Pre-exposure to PBO significantly increased the susceptibility of *Ae. aegypti* to deltamethrin and permethrin (*P* < 0.001). This indicates that in addition to *kdr* mutants, metabolic enzymes (monooxygenases) may be involved in the resistance phenotypes observed in the *Ae. aegypti* populations in these sites.

**Conclusion:**

Insecticide resistance underpinned by multiple mechanisms in *Ae. aegypti* indicates the need for surveillance to assist in developing appropriate vector control strategies for arboviral disease control in Ghana.

**Graphical Abstract:**

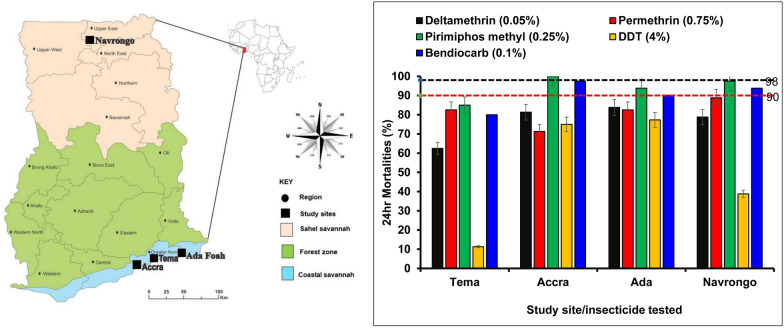

## Background

*Aedes*-borne arboviral diseases are a growing public health concern, but their control and prevention have received limited attention in Ghana in Africa [1]. It has been suggested that Africa could experience a shift in vector-borne diseases from malaria to arboviruses because of the effects of warming temperatures as a result of climate change [2]. Evidence for this comes from the growing number of arboviral outbreaks such as yellow fever and dengue fever reported in West Africa in the last 5 years [3–7]. Ghana has had a long history of yellow fever epidemics [8] with the most recent outbreak reported in October 2021 [9]. Recently, dengue virus was detected in suspected malaria and Ebola patients in Ghana [10, 11]. Furthermore, exposure to dengue and chikungunya virus has been established in Ghana via immunological surveys [12–14]. Despite such outbreaks and detections, there are major gaps in arboviral diseases and vector surveillance in West Africa [15].

*Aedes aegypti* is the main vector for yellow fever and dengue fever, whereas *Aedes albopictus* is an extremely invasive species and is spreading rapidly globally [16]. One or both of these vectors are commonly found in urban and suburban settings in Africa; however, their control receives limited attention [1, 17]. Control and prevention of arboviral diseases depend heavily on vector control using insecticides in combination with larval source reduction and case management. Pyrethroids are the predominant insecticides for vector control because of their low toxicity to humans and low cost. Thus, pyrethroids are commonly used for indoor and outdoor space spraying to control adult *Ae. aegypti* [18]. Intentional and inadvertent exposure to insecticides has caused mosquito populations to develop resistance through natural selection [19].

The spread of insecticide resistance in *Aedes* mosquitoes represents a major challenge for vector control strategies. Resistance of *Aedes* mosquitoes to insecticides has been reported in several West African countries including Burkina Faso, Cameroon, Senegal and Ghana [20–22]. In Ghana, resistance of *Aedes aegypti* to three of the classes of insecticides [pyrethroids, the organochlorine (DDT) and carbamates] recommended by WHO for vector control has been reported [23–25]. Mechanisms of resistance implicated in *Aedes* worldwide usually involve target-site mutations and metabolic detoxification [26].

Many target site knockdown resistance (*kdr*) mutations have been identified as resistance markers in *Aedes* mosquitoes globally [26–29]. So far, three *kdr* mutations have been detected in African *Aedes* populations, V410L, V1016I and F1534C [28, 30]. In Ghana, two of these mutations (V1016I and F1534C) have been found to cause resistance to pyrethroids [24, 28], with F1534C being the most common [31]. The *kdr* mutation V410L causes reduced sensitivity to pyrethroids [32] and was recently reported in *Ae. aegypti* from Burkina Faso and Cote d’Ivoire [33, 34].

The involvement of detoxification enzymes in resistance has been established by several studies in Africa, commonly via the use of synergists which elevate insecticide mortality [22, 35–38]. Piperonyl butoxide (PBO) is a synergist that primarily inhibits the cytochrome P450 monooxygenase superfamily of enzymes, members of which are frequently implicated in the metabolism of insecticides (especially pyrethroids) in mosquitoes [26]. Nets containing PBO-insecticide combinations (PBO nets) are now commonly distributed for malaria control, with demonstrated efficacy against *Anopheles* vector populations [39]. PBO has also been found to restore the susceptibility of several African *Aedes* populations to insecticides [20, 40, 41].

There is a need for a more effective arboviral vector control programme in response to the emergence of arboviral diseases in Africa. Surveillance of insecticide resistance in the target vector population is important to ensure rational choices for vector control strategies. Currently, there is a paucity of data on the insecticide resistance and mechanisms in *Aedes* mosquitoes in Ghana and Africa as a whole [1]. Here, we investigated the insecticide resistance status and mechanisms of *Ae. aegypti* mosquitoes in southern and northern Ghana to provide information for control.

## Materials and methods

### Study Sites

**Fig. 1:**
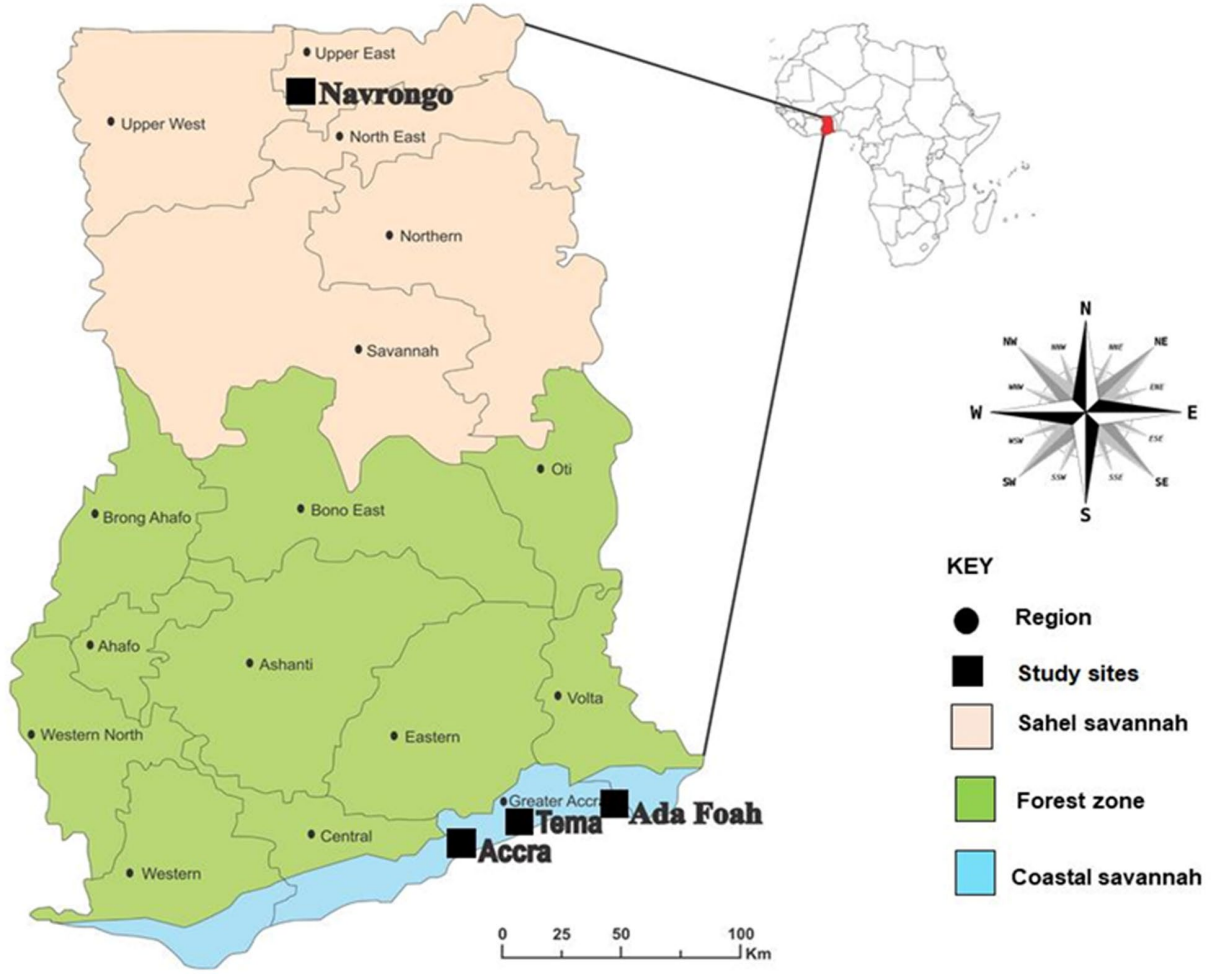
Map of Ghana showing the sites where *Aedes* mosquitoes were collected

The study was carried out in four sites in the southern and northern parts of Ghana, from which larval collections were made during the rainy and dry seasons from June 2019 to January 2020. The sites were Korle Bu, Accra (5° 33’ N, 0° 12’ W), Tema (5°40′0″N, 0°0′0″E), Ada Foah (5°47′N, 0°38′E) and Navrongo (10°53′5″N, 01°05′25″W) (Fig. 1).

Korle Bu, Tema and Ada are situated in the Greater Accra region in the southern part of Ghana. These sites are urban areas with an abundance of *Aedes* breeding sites and *Aedes* mosquitoes, which may increase the risk of arboviral transmission [25]. Tema is home to Ghana’s largest seaport where car tyres are imported, thus facilitating the importation of *Aedes* mosquitoes including invasive species such as *Ae. albopictus*. Navrongo is a town in the Sahel savannah zone of Northern Ghana, with a high risk of arboviral transmission due to its proximity to neighbouring Burkina Faso where recent dengue outbreaks have occurred [42].

### Larval Collection and Rearing

Immature forms of *Aedes* mosquitoes were collected from their breeding habitats—mainly abandoned car tyres, discarded containers and cans—within each of the study sites. *Aedes* larvae sampled were transported to the insectary at the Department of Medical Microbiology, University of Ghana Medical School, Accra, where they were raised to adults under stable conditions (temperature: 25 ± 2 °C, 80 ± 4% relative humidity). The larvae were fed on TetraMin Baby fish food (Tetra Werke, Melle, Germany). Emerged adults were fed on a 10% sugar solution until use in WHO susceptibility bioassays or synergist bioassays.

### Adult susceptibility testing

Susceptibility tests using WHO tubes were conducted according to the WHO protocol [43] to determine phenotypic resistance. Three- to 5-day-old female mosquitoes were exposed to papers impregnated with the pyrethroids permethrin (0.75%) and deltamethrin (0.05%), DDT (4%), the organophosphate pirimiphos-methyl (0.25%) and the carbamate bendiocarb (0.1%). Though these doses are not the recommended doses for evaluating the susceptibility of *Aedes* mosquitoes, they are the most commonly used [20, 25, 26]. These doses were used in the absence of WHO-recommended doses for *Aedes* mosquitoes at the time of the bioassay, which are currently 0.03%, 0.25% and 0.21% for deltamethrin, permethrin and pirimiphos-methyl respectively [44].

The knockdown time was recorded every 10 min during the 60-min exposure period. Mortality was recorded after a 24-h recovery period. Alive (resistant) and dead (susceptible) mosquitoes were stored in absolute ethanol for later DNA analysis.

### Morphological Species Identification

Resistant and susceptible *Aedes* mosquitoes from all WHO susceptibility bioassays were morphologically identified using identification keys by Huang [45].

### Genotyping of *kdr* mutations in *Aedes aegypti* populations

A subsample of 332 *Aedes* mosquitoes that were phenotypically resistant and susceptible to insecticides deltamethrin, permethrin and DDT from the bioassay tests were randomly selected for genotyping of *kdr* mutations, F1534C, V1016I and V410L. A total of 172 resistant *Aedes* mosquitoes and 160 susceptible mosquitoes representing mosquitoes from all the four study sites were used for the genotyping. Total DNA was extracted from whole mosquitoes using the DNeasy Tissue Kit (Qiagen, USA). Pyrethroid and DDT-resistant and -susceptible *Ae aegypti* were genotyped for *kdr* mutations, F1534C, V1016I and V410L, using allele-specific PCR according to the protocols of Linns et al. [46] and Villanueva-Segura et al. [47]. Primer sequences are shown in Table 1.

**Table 1:** List of primer sequences used for detecting allele-specific *kdr* mutations in the voltage-gated sodium channel gene of *Aedes* mosquitoes

*kdr* mutation	Primers	Sequence (5' -3')	References
V1016I	1016 Val ^+ ^(for)	## ACAAATTGTTTCCCACCCGCACCGG	[46]
	1016 Ile kdr (for)	#ACAAATTGTTTCCCACCCGCACTGA	
	1016 common (rev)	GGATGAACCGAAATTGGACAAAAGC	
F1534C	1534 Phe + (for)	#TCTACTTTGTGTTCTTCATCATATT	[46]
	1534 Cys kdr (for)	##TCTACTTTGTGTTCTTCATCATGTG	
	1534 common (rev)	TCTGCTCGTTGAAGTTGTCGAT	
	long 5'-tail	GCGGGCAGGGCGGCGGGGGCGGGGCC	
	short 5'-tail	GCGGGC	
V410L	V410fw	GAT AAT CCA AAT TAC GGG TAT AC	[47]
	V410fw [L – GC]	ATC TTC TTG GGT TCG TTC TAC CGT G	
	L410fw [S – GC]	ATC TTC TTG GGT TCG TTC TAC CAT T	
	410rev	ATC TTC TTG GGT TCG TTC TAC CAT T	
	[L – GC]	GCG GGC AGG GCG GCG GGG GCG GGG CC	
	[S – GC]	GCG GGC	

^+^ wild-type specific primer, *kdr* kdr specific primer, ^#^short 5′tail attached, ^##^long 5′tail attached, *fw* forward primer, *rev* reverse primer

### Synergist assays with PBO

Piperonyl butoxide (PBO) synergist assays were performed to establish the role of cytochrome P450s in the observed resistance of *Aedes* mosquitoes. This synergist assay was performed using WHO tubes and papers, with four replicates of 20 female *Aedes* mosquitoes each pre-exposed to 4% PBO-impregnated papers for 1 h, after which the mosquitoes were immediately exposed to deltamethrin (0.05%) or permethrin (0.75%) for another 1 h. For each test, two control tubes with 20 female mosquitoes each were set up, one with PBO alone papers and the other with oil-impregnated papers. The two control tubes were included in the set-up for testing. Knockdown was recorded during the 60 min period and mortality after 24 h. The synergist assays were performed according to WHO criteria [48].

### Statistical analysis

WHO insecticide susceptibility tests and PBO synergist tests were analyzed using the WHO criteria [43]. Mosquitoes were classified as susceptible if the mortality rate was between 98 and 100%; as suspected resistant if the mortality rate was between 90 and 97%; as resistant if the mortality rate was < 90% [43]. Generalised linear models with binomial link function (in SPSS 26) were used to compare bioassay mortalities for each insecticide among study sites, with overall Wald Chi-square analysis results shown and populations showing differences indicated. The Chi-square test or Fisher’s exact tests were used in determining associations between *kdr* mutations and phenotypes in genotypic and allelic tests, with odds ratios used to measure effect size. Probability values < 0.05 were interpreted as statistically significant.

## Results

### Morphological species identification of resistant and susceptible *Aedes* mosquitoes

A sub-sample of 409 *Aedes* mosquitoes obtained through random sampling from a total of 2240 mosquito samples that were used for the bioassays were used for morphological identification using taxonomic keys. All the 237 *Aedes* mosquitoes from Korle-bu, Tema and Accra in southern Ghana and 172 *Aedes* mosquitoes from northern Ghana that were morphologically identified were found to be *Ae. aegypti* (100%).

### Phenotypic resistance

**Fig. 2.**
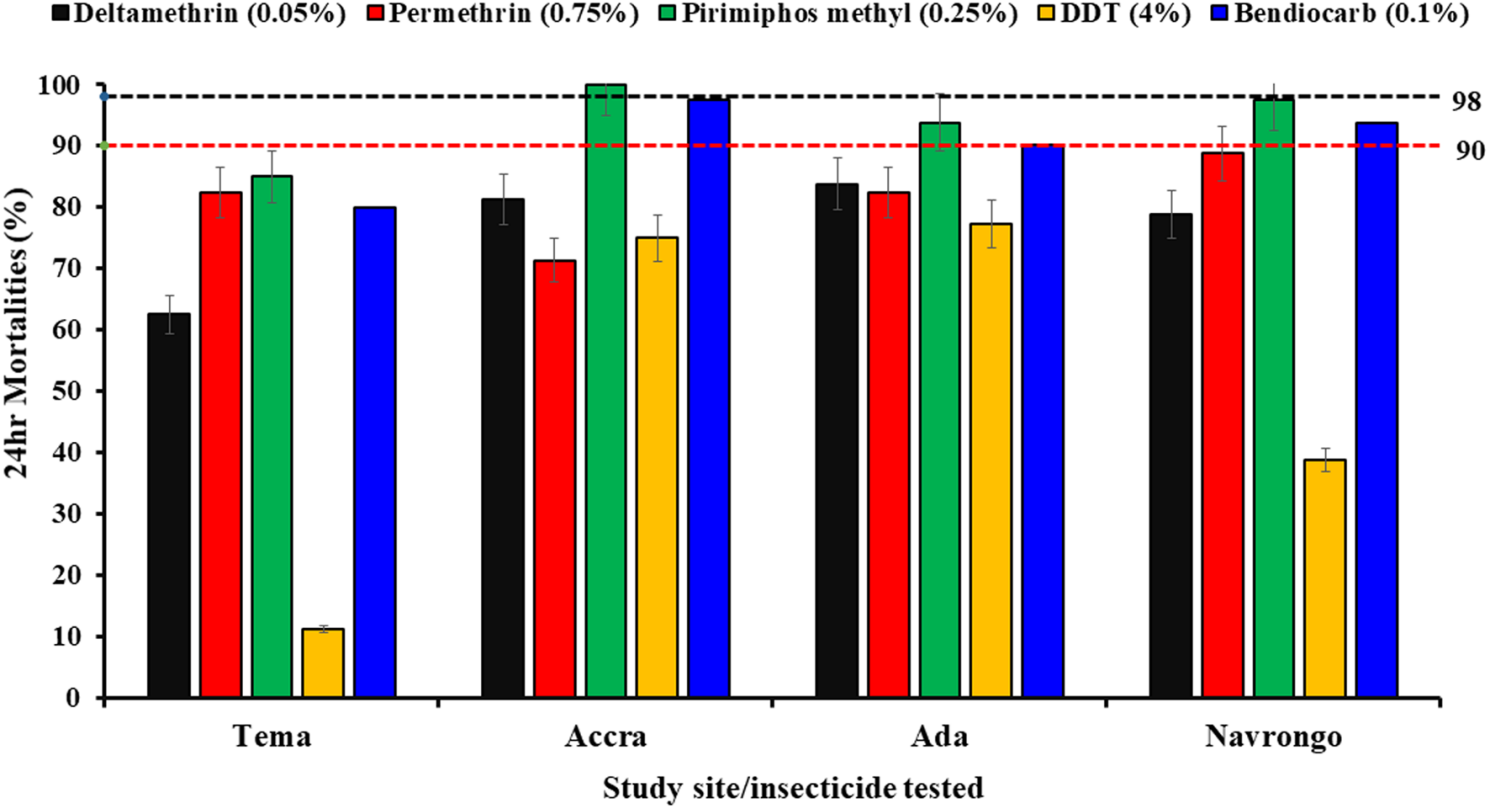
Twenty-four-hour mortalities of *Aedes* mosquitoes with exposure to insecticides, permethrin, deltamethrin, DDT, pirimiphos-methyl and bendiocarb. Error bars represent the 95% confidence interval of the mean

Mortality of *Aedes* mosquitoes to DDT was significantly lower in Tema (11.7%) than in Navrongo (38.8%), Ada (77.3%) and Accra (75%), which were similar (*χ2* = 77.493, *df* = 3, *P* < 0.001). Resistance to permethrin was also detected in each site: Tema (82.5%), Accra (71.3%), Ada Foah (82.5%) and Navrongo (88.8%), though with much more limited variability (*χ*^*2*^ = 8.024, *df* = 3, *P* = 0.046). Mortality rates to deltamethrin were significantly lower in the population from Tema (62.5%) compared to the other sites Accra (81.3%), Ada Foah (83.8%) and Navrongo (78.8%) (*χ*^*2*^ = 11.826, *df* = 3, *P* = 0.008). Bendiocarb resistance was found in Tema (80%) and was significantly higher than the mortalities in Accra (97.5%) and Navrongo (93.8%) and marginally vs. Ada (90.1%), each of which is classified as suspected resistant (*χ*^*2*^ = 13.014, *df* = 3, *P* = 0.005). Mosquitoes were resistant to pirimiphos-methyl in Tema (85%) but showed suspected resistance in Ada Foah (93.8%) and Navrongo (97.5%) whilst being susceptible in Accra (100%), with significant but relatively moderate variation among the sites (*χ*^*2*^ = 7.582, *df* = 3, *P* = 0.023) (Fig. 2).

### Genotyping of *kdr*-resistant mutations and their association with phenotypic resistance

A subset of 332 *Ae. aegypti* obtained from the phenotypic assays were genotyped for the F1534C, V1016I and V410L *kdr* mutations. The genotypes and allele frequencies of each *kdr* mutation are shown in Table 2. The 1534C *kdr* mutation was detected with a high allelic frequency of 1 in the pyrethroid and DDT-resistant mosquitoes and 0.65 to 1 in the susceptible group. No significant association was observed between the presence of F1534C mutation and resistant phenotypes (Table 3). The V1016L mutation was also detected in all the sites with allelic frequencies ranging from 0.87 to 0.97 in the resistant group and 0.65 to 0.91 in the susceptible group. The V1016I mutation was significantly associated with permethrin resistance (OR = 13.2, 95% CI = 2.8–122, *P* < 0.001) (Table 3).

**Table 2:** Number of genotypes and frequencies of *kdr* mutations in the VGSC gene of *Aedes aegypti* mosquitoes

Insecticide	Study site	Phenotype	n	F1534C				V1016I				V410L			
				CC	FC	FF	Allele Freq	II	VI	VV	Allele Freq	LL	VL	VV	Allele Freq
Deltamethrin	Tema	R	21	21	0	0	1	20	0	1	0.95	4	1	16	0.08
		S	20	20	0	0	1	14	1	5	0.73	7	1	12	0.09
	Ada	R	9	9	0	0	1	9	0	0	1	0	0	10	0
		S	20	19	0	1	0.95	19	1	0	0.98	0	2	18	0.05
	Navrongo	R	9	9	0	0	1	7	0	2	0.78	1	0	8	0.11
		S	20	20	0	0	1	16	4	0	0.90	0	0	20	0
	Accra	R	14	14	0	0	1	14	0	0	1	0	0	14	0
		S	20	20	0	0	1	17	3	0	0.93	0	0	20	0
	Total	R	53	53	0	0		52	9	3		5	1	48	
		S	80	79	0	1		66	9	5		7	3	70	
Permethrin	Tema	R	15	15	0	0	1	14	1	0	0.97	4	1	10	0.30
		S	20	18	0	2	0.9	13	1	6	0.68	2	1	17	0.13
	Ada	R	10	10	0	0	1	8	0	2	0.80	2	0	8	0.20
		S	20	19	0	1	0.95	5	3	12	0.33	0	0	20	0
	Navrongo	R	20	20	0	0	1	20	0	0	1	6	3	11	0.38
		S	20	19	0	1	0.95	17	3	0	0.93	0	0	20	0
	Accra	R	14	14	0	0	1	14	0	0	1	0	0	14	0
		S	20	6	14	0	0.65	3	17		0.58	0	0	20	0
	Total	R	59	59	0	0		56	1	2		12	4	43	
		S	80	62	14	4		38	24	18		2	1	77	
DDT	Tema	R	14	14	0	0	1	10	4	0	0.86	4	0	10	0.29
		S	–	–	–	–	–	–	–	–	–	–	–	–	–
	Ada	R	12	12	0	0	1	10	0	2	0.83	1	1	10	0.13
		S	–	–	–	–	–	–	–	–	–	–	–	–	–
	Navrongo	R	26	26	0	0	1	26	0	0	1	8	3	15	0.37
		S	–	–	–	–	–	–	–	–	–	–	–	–	–
	Accra	R	8	8	0	0	1	8	0	0	1	0	0	8	0
		S	–	–	–	–	–	–	–	–	–	–	–	–	–
	Total	R	60	60	0	0		54	4	2		13	4	43	

*VV* Wild type (susceptible), *VL* heterozygotes, *LL* mutant (resistant), *VI* heterozygotes, *II* mutant (resistant), *FF* wild type (susceptible), *FC* heterozygotes, *CC* mutant (resistant); *n* sample size, – not genotyped

**Table 3:** Distribution of *kdr* alleles and its association with phenotypes across study sites and insecticides

Site	Pheno-type	F1534C			*P*	OR (95% CI)		V1016I			*P*	OR (95% CI)		V410L			*P*	OR (95% CI)	
		C C	FC	FF		CC/ FF	FC/ FF	I I	VI	VV		II/ VV	VI/ VV	LL	VL	VV		LL/ VV	VL/ VV
Tema	R	50	0	0	0.19	nd	nd	44	5	1	0.00^*^	17.9 (2.3- 778.5	27.5(1.4- 1413.2	12	2	36	1.0	1.07 (0.3–3.3)	0.81 (0.005- 11.8)
	S	38	0	2				27	2	11				9	2	29			
Ada	R	31	0	0	0.50	nd	nd	27	0	4	0.02^*^	3.4(0.85–16)	0 (0- 3.68)	2	1	28	0.30	nd	0.68 (0.01- 13.7)
	S	38	0	2				24	4	12				0	2	38			
Navrongo	R	54	0	1	1.0	1.38 (0.017–110.6)	nd	53	0	2	0.00^*^	0 (0 -3.2)	0 (0- 0.23)	14	6	35	0.00 ^*^	nd	nd
	S	39	0	1				33	7	0				0	0	40			
Accra	R	36	0	0	0.00	nd	nd	35	0	1	0.00^*^	nd	nd	0	0	36	nd	nd	nd
	S	26	14	0				20	20	0				0	0	40			
Insecticides																			
Deltamethrin	R	85	0	0	0.62	nd	nd	80	9	3	1.0	1.12 (0.2–7.5)	1.66(0.2–13.8)	5	1	48	1.0	1.04(0.2–4.1)	0.48(0.009–6.29)
	S	139	0	1				118	9	5				7	3	70			
Permethrin	R	59	0	0	0.07	nd	nd	56	1	2	0.00^*^	13.20(2.8–122)	0.375(0.006–7.9)	12	4	43	0.49	2.39(0.4–23.8)	7.162(0.7–357)
	S	62	14	4				38	24	18				2	1	77			

*VV* wild type (susceptible), *VL* heterozygotes, *LL* mutant (resistant), *VI* heterozygotes, *II* mutant (resistant), *FF* wild type (susceptible), *FC* heterozygotes, *CC* mutant (resistant), *DDT* dichlorodiphenyltrichloroethane, *nd* not determined, *P* Fisher’s exact *P*-value, *significant (*P* < 0.05), *CI* confidence interval

The predominant genotype was the homozygote mutant genotype for the 1534C and 1016I mutation (Table 2). The allele frequency for the V410L *kdr* mutation varied between 0 and 0.38 depending on the insecticide, collection site and whether dead or alive (Table 2). There was no significant association between the V410L mutation and mortality with either insecticide pooled across study sites, whilst for pooled insecticides, there was a significant association only in Navrongo (Table 3).

### Triple-locus *kdr* frequencies and phenotypic associations

**Fig. 3:**
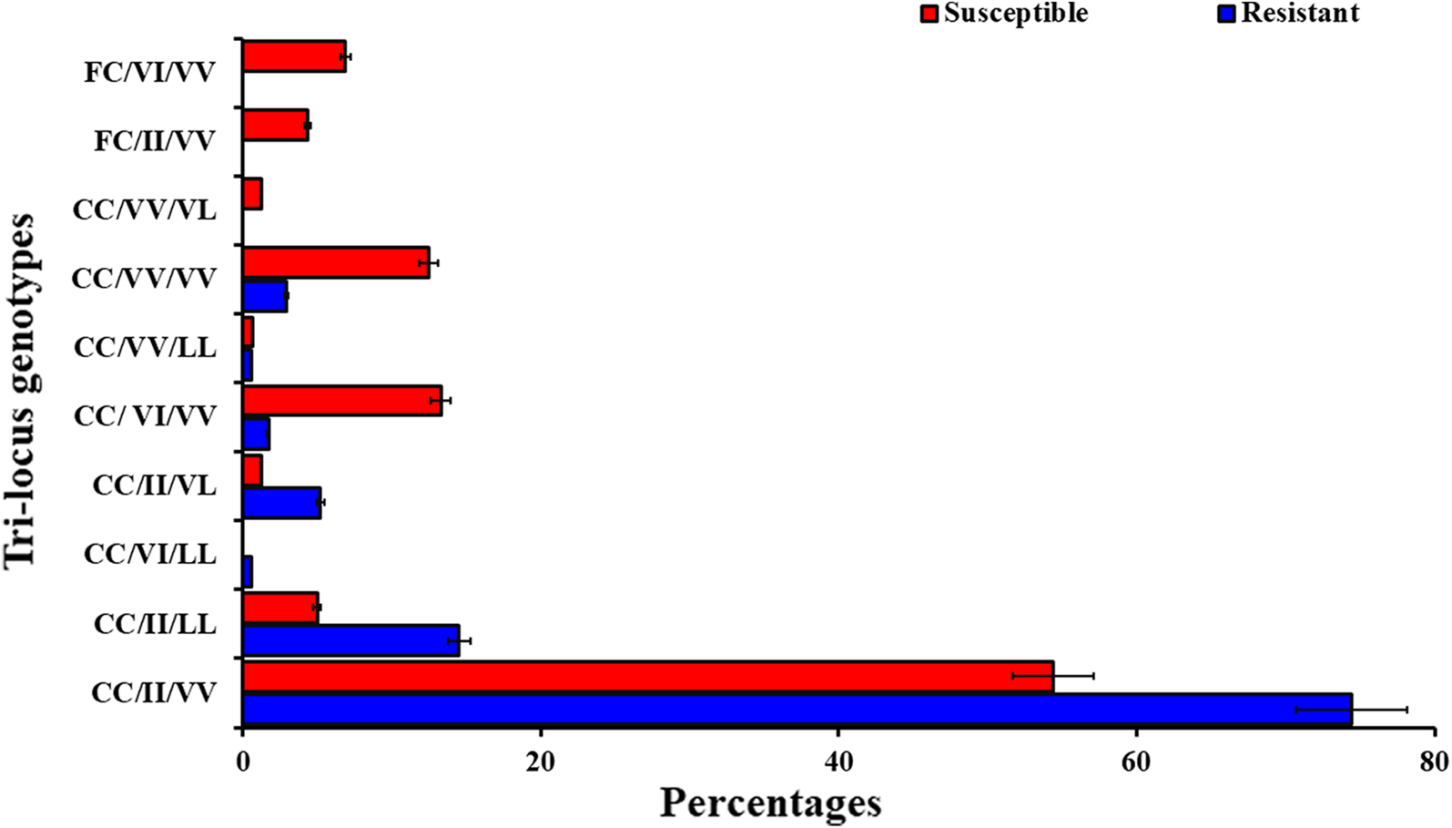
Frequencies of tri-loci genotypes for the VGSC mutations in phenotyped *Aedes aegypti* mosquitoes. Each tri-locus genotyped is named according to the genotypic composition at each *kdr* mutation following the order 410 (VV, VL or LL)/1016 (VV, VI or II)/1534 (FF, FC or CC). VV, wild type (susceptible); VL, heterozygotes; LL, mutant (resistant); VI, heterozygotes; II, mutant (resistant); FF, wild type (susceptible); FC, heterozygotes; CC, mutant (resistant)

Ten genotypes were observed out of a total of 27 possible genotype combinations across the three *kdr* loci in the 332 mosquitoes genotyped (Fig. 3). The most common tri-locus genotype detected across all sites was the homozygote mutant for F1534C (CC) and V1016I (II) combined with the homozygote wild type for V410L (VV). This tri-loci genotype (CC/II/VV) was detected in 128 (74.4%) resistant and 87 (54.4%) susceptible *Ae. aegypti* mosquitoes across all the sites. The triple homozygote mutant CC/II/LL was present in 25 (14.5%) resistant and 8 (5%) susceptible *Ae. aegypti* mosquitoes (Fig. 3).

Of the three most common tri-locus genotypes, CC/II/VV and CC/II/LL were significantly associated with permethrin resistance with a fivefold (OR = 5.96, 95% CI = 2.6–13.7, *P* < 0.001) and sevenfold (OR = 7.02, 95% CI = 1.3–68.5, *P* < 0.05) greater likelihood of resistance respectively (Table 4). No significant association with deltamethrin resistance was observed in the tri-loci genotypes, CC/II/VV, CC/II/LL and CC/VV/LL (*P* > 0.05) (Table 4).

**Table 4:** Distribution of tri-loci genotypes and their genetic association with insecticide resistance phenotypes

Insecticide	phenotype	CC/II/VV	OR(95% CI	*P*	CC/II/LL	OR(95% CI	*P*	CC/VV/VV	OR(95% CI	*P*
Permethrin	R	45	5.96(2.6- 13.7)	0.000^*^	9	7.02(1.3–68.5)	0.008*	0	nd	nd
	S	28			2			0		
Deltamethrin	R	44	1.97(0.8–5.3)	0.148	5	1.28(0.3- 5.3)	0.68	3	1.54(1.97- 11.92)	0.68
	S	57			6			3		
DDT	R	39	na		12	na	Na	1	na	na
	S	Na								

Each tri-locus genotyped is named according to the genotypic composition at each *kdr* mutation following the order 410 (VV, VL or LL)/1016 (VV, VI or II)/1534 (FF, FC or CC). *VV* wild type (susceptible); *LL* mutant (resistant); *II* mutant (resistant); *CC* mutant (resistant), *OR* odds ratio, *CI* confidence interval, *DDT* dichlorodiphenyltrichloroethane, *nd* not determined, *na* not applicable because the group was not genotyped, *P* *P*-value (Fisher’s exact), *P < 0.05(significant). *CI* Confidence interval

To analyse the relationship between the number of *kdr* alleles across the three loci and resistance phenotypes for each insecticide, three categories were created based on comparable frequencies of each: 1–3 *kdr* alleles; 4 *kdr* alleles; 5–6 *kdr* alleles. Generalised linear model analysis revealed a strong relationship between the number of *kdr* alleles and survival to permethrin, with 5–6 *kdr* alleles conferring significantly greater resistance than both 1–3 alleles (OR = 114.3, *P* <  0.001) and 4 *kdr* alleles (OR = 4.8, *P*− 0.047). However, though deltamethrin mortality was the highest in the 1–3 allele category (0.78 vs. 0.57 for both of the other categories), the difference was not significant, indicating that resistance was not dependent on the number of *kdr* alleles.

### Synergist assays

**Fig. 4:**
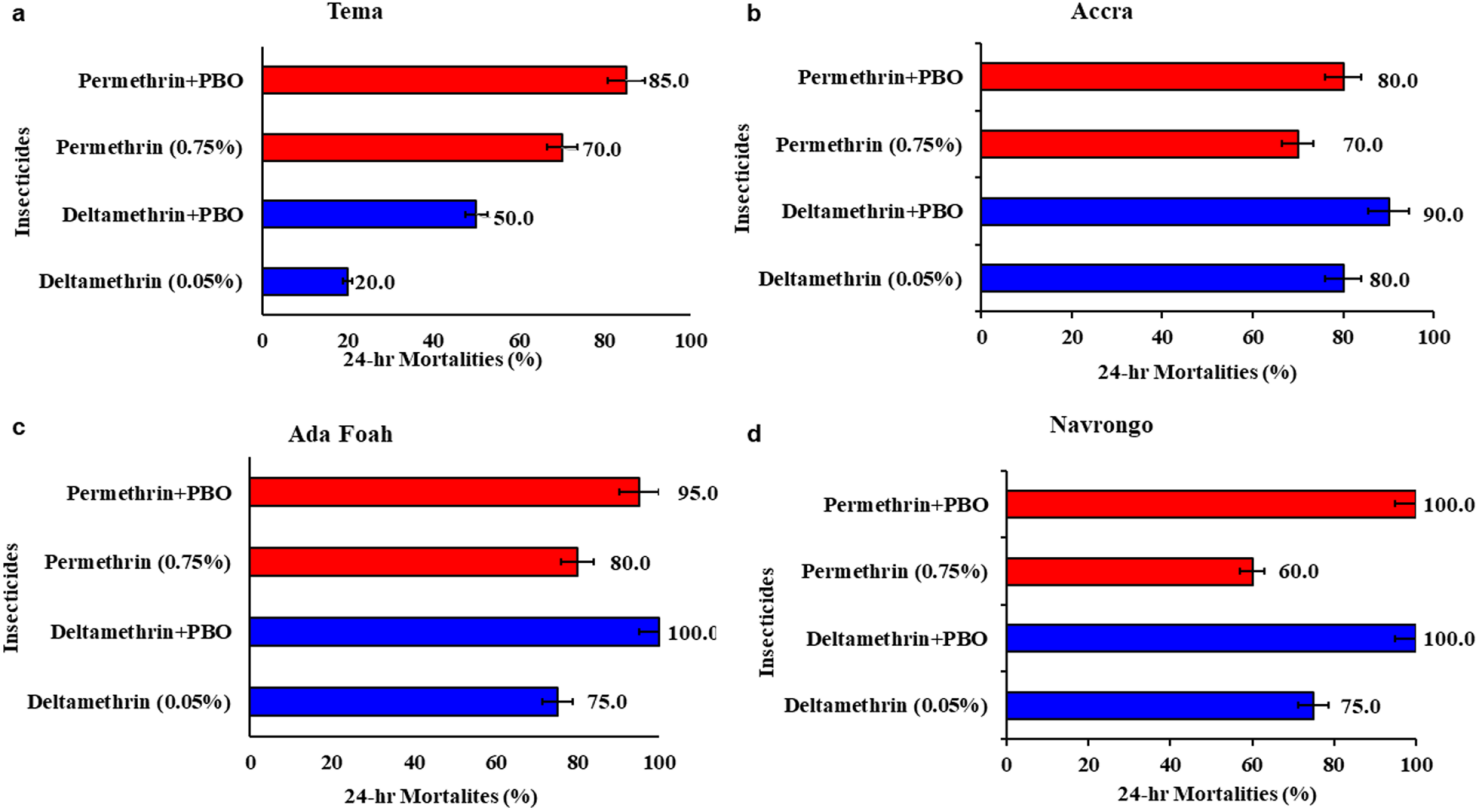
Synergistic effects of PBO on the insecticide susceptibility status of *Aedes* populations from study sites. **a**–**d** The 24-h mortalities of *Aedes* mosquitoes from Tema (**a**), Accra (**b**), Ada (**c**) and Navrongo (**d**) respectively. Error bars represent the 95% confidence interval of the mean

Piperonyl butoxide (PBO) increased the susceptibility of *Ae. aegypti* to pyrethroids across the sites and insecticides (*χ*^*2*^ = 26.100, *df* = 3, *P* < 0.001; GLM interaction terms involving site and insecticide with PBO each non-significant). Mosquitoes from Tema had an increase in mortality rates to deltamethrin (from 20 to 50%) and permethrin (from 70 to 85%) after PBO exposure (Fig. 4a). Pre-exposure of *Ae. aegypti* from Accra increased the mortality rates to deltamethrin (from 80 to 90%) and permethrin (70% to 80%) **(**Fig. 4b**)**. For Ada Foah, synergist-insecticide combinations reversed permethrin resistance in *Ae. aegypti* from 75 to 100% while partial susceptibility restoration was observed with deltamethrin 80% to 95% (Fig. 4c). Similarly, pre-exposure of *Ae. aegypti* mosquitoes from Navrongo to PBO showed full recovery of susceptibility to permethrin (from 60 to 100%) and deltamethrin (from 75 to 100%) (Fig. 4d). PBO has a significant effect on mortality of *Ae. aegypti* to pyrethroids deltamethrin and permethrin. Overall, PBO increased the mortality from 0.68 to 0.89 (OR = 4.1; *P* < 0.001).

## Discussion

This study provides evidence of the resistance of *Ae. aegypti* populations in Ghana to public health insecticides. Females were resistant to DDT and pyrethroids, deltamethrin and permethrin in all the study sites. Knockdown resistance mutations F1534C and VI016I were at high frequencies, whilst the V410L *kdr* mutation was present at lower frequencies in Tema, Ada and Navrongo. Increased mortality to both pyrethroids was observed in *Ae. aegypti* in all sites after pre-exposure to PBO.

All mosquitoes that were randomly sampled for morphological identification were found to be *Ae. aegypti*. These findings are similar to that of another study in Ghana, where the most predominant species in urban and suburban sites was *Ae. aegypti* [25] and generally more likely to be found in urban and suburban areas [1, 49]. *Aedes aegypti* was the most common species across six regions in Ghana based on surveillance data obtained from 2015 to 2016 by Amoa-Bosompem et al. [50]. Also, *Ae. aegypti* was the predominant species (75.5%) in an urban site, Accra, according to a study by Suzuki et al. [23]. Therefore, multiple studies enable the conclusion that *Ae. aegypti* is the dominant vector in urban and suburban areas in Ghana.

Overall, the resistance profile of *Ae. aegypti* mosquitoes to major insecticides used for public health varied across study sites. Pyrethroids and DDT resistance in *Ae. aegypti* populations were widespread across all the sites. Evidence of pyrethroid resistance in *Ae. aegypti* was also established in other previous studies from Ghana [23–25] and other African countries [20, 21, 28]. However, what is driving insecticide resistance in these populations is uncertain. This is because current vector control measures in Ghana involve the use of IRS and LLINs, which are mainly targeting indoor resting mosquitoes. Previous studies in West Africa have shown that *Ae. aegypti* mosquitoes tend to rest outdoors so are not likely to have as many IRS and LLINs encounters [25, 42]. The extent of the involvement of these measures on resistance in Ghanaian *Ae. aegypti* population is largely unknown. Thus, calls are being made for more studies on the mediators of insecticide resistance in *Ae. aegypti* populations to be better equipped for arboviral vector control in Ghana.

Also, resistance to bendiocarb was observed in Tema while suspected resistance to bendiocarb was also observed in the other sites. An earlier study on *Aedes* mosquitoes in Ghana showed suspected resistance and susceptibility to bendiocarb in Ghanaian *Aedes* populations [25]. This provides evidence that bendiocarb resistance is increasing in *Aedes* populations. Other studies from Burkina Faso, Cameroon and Cote d’Ivoire also reported bendiocarb resistance in *Ae. aegypti* populations [33, 41, 51]. Our findings also showed resistance and suspected resistance to pirimiphos-methyl in all sites except in Accra, where it was susceptible. Other studies in Ghana and West Africa have reported susceptibility of *Ae. aegypti* populations to organophosphate insecticides [25, 41, 51]. However, our findings and those of other studies from Cote d’Ivoire and Senegal with recent evidence show that organophosphate resistance is also increasing [22, 33]. This calls for more surveillance of organophosphate and carbamate resistance in Ghanaian *Ae. aegypti* populations.

In this study, high frequencies of the F1534C and V1016I mutations were detected in both resistant and susceptible *Ae. aegypti* mosquitoes genotyped. Previous studies in Ghanaian *Ae. aegypti* in 2016 also detected high frequencies on the F1534C mutation and one heterozygote mutation of the V1016I mutation [28]. It is alarming to observe an increase in the frequency of V1016I to the point of nearing fixation in some of the study sites as well as the detection of the V410L mutation in Ghanaian *Ae. aegypti* populations. Similarly, the F1534C mutation has been found to be nearly fixed in *Ae. aegypti* mosquitoes from Cameroon (90%) and Burkina Faso (97%) [35, 52]. Relatively low allelic frequencies of the V410L mutation were observed across the study sites in both the resistant and susceptible groups of *Ae. aegypti* mosquitoes genotyped. This is the first report to our knowledge of this mutation in the northern part of Ghana, Navrongo. It was first reported in the southern part of Ghana, Accra, in only forest populations in 2022 [53]. This mutation was first detected in a Brazilian *Ae. aegypti* strain in 2017 [32]. It was detected in high frequencies in Angola (0.83), and low frequencies in Portugal (0.17) and Cote d’Ivoire (0.28) [32, 33]. These *kdr* mutations were found to be significantly associated with permethrin resistance. However, no significant association was observed between the *kdr* mutations and deltamethrin resistance. This is contrary to in vitro work by Haddi et al. [32] where both permethrin and deltamethrin resistance was significantly associated with the presence of the 410L allele. This finding of a limited impact of the 410L mutation on deltamethrin resistance was also evident in the analysis of the relationship between the number of *kdr* alleles and survival; the contrast was extremely strong for permethrin, especially for the 5–6 *kdr* allele category, all of which harboured 410L mutants.

Findings from this study revealed an increase in the mortality of *Ae. aegypti* mosquitoes to pyrethroids, deltamethrin and permethrin after pre-exposure to PBO. There was a significant increase in the mortality rates of *Ae. aegypti* mosquitoes after pre-exposure to PBO across all the sites. In sites Ada Foah and Navrongo, total restoration of susceptibility was observed after pre-exposure to PBO. Similar findings have been observed in Cameroon [21] and in Nigeria [37], where the mortality rate to pyrethroids was increased after pre-exposure to PBO synergist. Results obtained for PBO assays are useful for arboviral vector control, especially in endemic areas with high resistance among the vector populations. PBO can be incorporated in insecticide combinations to help increase the mortality of resistant *Ae. aegypti* mosquitoes to pyrethroids. The increase in mortality and restoration of susceptibility observed after PBO exposure confirms the role of monooxygenases in pyrethroid resistance that was observed. Therefore, we recommend that further studies should be done to identify the specific monooxygenases such as cytochrome P450s involved in pyrethroid resistance in *Ae. aegypti* populations in Ghana.

## Conclusion

This study shows moderate to high phenotypic resistance among *Ae. aegypti* populations across the study sites. Knockdown resistance mutations F1534C and V1016I were found in high frequencies in *Ae. aegypti* populations across the study sites while V410L mutation was also detected in low frequencies. Pre-exposure of *Ae. aegypti* mosquitoes to PBO increased their mortalities to the pyrethroid insecticides tested. It is important to determine the intensity of resistance in *Ae. aegypti* populations in Ghana and also look into the possibility of adapting an integrated approach using newer classes of insecticides, larval source management, mass trapping and biological control toward the control of *Aedes* mosquitoes in Ghana [54].

## Data Availability

All datasets generated and/or analyzed during this study are included in the manuscript.

## Abbreviations

WHO: World Health Organization
PBO: Piperonyl butoxide
VGSC: Voltage-gated sodium channel
*kdr*: Knockdown resistance
V: Valine
G: Glycine
I: Isoleucine
C: Cysteine
F: Phenylalanine
L: Leucine
DDT: Dichlorodiphenyltrichloroethane
GST's: Glutathione S-transferase
CYPs: Cytochrome P450s
*Ae.*: *Aedes*
UV: Ultraviolet
IRS: Indoor residual spraying
LLINs: Long-lasting insecticide-treated nets
